# Tuberculosis Diagnosis: Current, Ongoing, and Future Approaches

**DOI:** 10.3390/diseases12090202

**Published:** 2024-09-03

**Authors:** Guilherme Bartolomeu-Gonçalves, Joyce Marinho de Souza, Bruna Terci Fernandes, Laís Fernanda Almeida Spoladori, Guilherme Ferreira Correia, Isabela Madeira de Castro, Paulo Henrique Guilherme Borges, Gislaine Silva-Rodrigues, Eliandro Reis Tavares, Lucy Megumi Yamauchi, Marsileni Pelisson, Marcia Regina Eches Perugini, Sueli Fumie Yamada-Ogatta

**Affiliations:** 1 Programa de Pós-Graduação em Fisiopatologia Clínica e Laboratorial, Universidade Estadual de Londrina, Londrina CEP 86038-350, Paraná, Brazil; guilherme.bartolomeu@uel.br (G.B.-G.); marsileni@uel.br (M.P.); marciaperugini@uel.br (M.R.E.P.); 2 Programa de Pós-Graduação em Microbiologia, Universidade Estadual de Londrina, Londrina CEP 86057-970, Paraná, Brazil; joycemarinhodesouza@gmail.com (J.M.d.S.); terci.bruna@gmail.com (B.T.F.); lais.spoladori@gmail.com (L.F.A.S.); guilhermeferreiracorreia@gmail.com (G.F.C.); isabela.mcastro@uel.br (I.M.d.C.); paulo.guilhermeph@uel.br (P.H.G.B.); gislaine.srodrigues@uel.br (G.S.-R.); tavares.eliandro@uel.br (E.R.T.); lionilmy@uel.br (L.M.Y.); 3 Faculdade de Ciências da Saúde, Biomedicina, Universidade do Oeste Paulista, Presidente Prudente CEP 19050-920, São Paulo, Brazil; 4 Curso de Farmácia, Faculdade Dom Bosco, Cornélio Procópio CEP 86300-000, Paraná, Brazil; 5 Departamento de Medicina, Pontifícia Universidade Católica do Paraná, Campus Londrina CEP 86067-000, Paraná, Brazil

**Keywords:** pulmonary tuberculosis diagnosis, *Mycobacterium tuberculosis*, diagnostic techniques, molecular techniques

## Abstract

Tuberculosis (TB) remains an impactful infectious disease, leading to millions of deaths every year. *Mycobacterium tuberculosis* causes the formation of granulomas, which will determine, through the host–pathogen relationship, if the infection will remain latent or evolve into active disease. Early TB diagnosis is life-saving, especially among immunocompromised individuals, and leads to proper treatment, preventing transmission. This review addresses different approaches to diagnosing TB, from traditional methods such as sputum smear microscopy to more advanced molecular techniques. Integrating these techniques, such as polymerase chain reaction (PCR) and loop-mediated isothermal amplification (LAMP), has significantly improved the sensitivity and specificity of *M. tuberculosis* identification. Additionally, exploring novel biomarkers and applying artificial intelligence in radiological imaging contribute to more accurate and rapid diagnosis. Furthermore, we discuss the challenges of existing diagnostic methods, including limitations in resource-limited settings and the emergence of drug-resistant strains. While the primary focus of this review is on TB diagnosis, we also briefly explore the challenges and strategies for diagnosing non-tuberculous mycobacteria (NTM). In conclusion, this review provides an overview of the current landscape of TB diagnostics, emphasizing the need for ongoing research and innovation. As the field evolves, it is crucial to ensure that these advancements are accessible and applicable in diverse healthcare settings to effectively combat tuberculosis worldwide.

## 1. Introduction

Tuberculosis (TB) is a bacterial disease caused by *Mycobacterium tuberculosis* (*sensu stricto*) and other members of the *Mycobacterium tuberculosis* complex (MTBC) [1,2]. The disease caused 1.3 million deaths in 2022 [3], with over 31 million TB-related deaths estimated for the coming years [4].

The lungs are the most commonly affected organs (pulmonary tuberculosis, PTB) [5]. However, the disease can affect other sites, such as pleura, lymph nodes, abdomen, genitourinary tract, skin, joints, bones, and meninges, a condition known as extrapulmonary TB [6], which afflicts between 15 and 20% of TB patients, especially HIV-positive individuals [7].

Typical symptoms of PTB include persistent cough, hemoptysis, chest discomfort, fatigue, weight loss, night sweats, and fever, although mild or no symptoms may occur in the initial stages [8,9]. Moreover, individuals with latent tuberculosis infection (LTBI) exhibit no active TB signs [10] and constitute a potential source for future active TB cases [11].

Transmission, mainly through cough-generated aerosols [12], is influenced by environmental conditions, microbial viability, and host immune response [13]. Risk factors include alcohol abuse [14], coinfection with HIV [15], anemia [16], diabetes [17], silicosis [18], smoking and exposure to air pollution [19], homelessness [20], and incarceration [21].

Upon infection, *M. tuberculosis* triggers the formation of granulomas [2,22], after which the pathogen can remain in a latent state or lead to active disease depending on the host’s immune response [2,23]. Since recent studies suggest a potential association between PTB and lung cancer, effective TB control is needed to reduce this risk [24,25,26].

The diagnosis of TB encompasses difficulties such as underdiagnosis and limited access to testing [27], a scenario that was worsened by the COVID-19 pandemic, although the last Global Tuberculosis Report signals some progress in reversing its impact on TB control [3]. Additionally, the growing incidence of non-tuberculous mycobacterial (NTM) infections, partially due to the aging of the population, as well as an increase in immunosuppressed individuals [28], poses challenges for the diagnosis of TB. In fact, non-tuberculous mycobacteria share some features with *M. tuberculosis*, and non-tuberculous pulmonary disease usually presents with nonspecific signs and radiological findings similar to those caused by the TB causative agent. Therefore, diagnosing non-tuberculous mycobacterial pulmonary disease in regions with high TB prevalence is a complex endeavor. Recent reviews have outlined methods for diagnosing NTM, and the reader is referred to these reviews for a greater understanding of the methods that are used or are being developed for the diagnosis of NTM [29,30]. We briefly summarized the main techniques, and they are shown in Supplementary Table S1 [31,32,33,34,35,36,37,38,39,40,41,42,43,44,45].

Early TB diagnosis is critical for global health since it prevents TB transmission [46,47,48] and reduces mortality, especially for people living with HIV [49]. Rapid diagnostic techniques enable prompt treatment initiation [50,51], aligning with the End TB Strategy, which emphasizes effective diagnosis and treatment for people with TB [52].

Therefore, this non-systematic literature review discusses the diagnosis of tuberculosis, particularly PTB, and identifies future research directions regarding TB diagnostics.

## 2. Traditional Diagnostic Methods

### 2.1. Sputum Smear Microscopy

Sputum smear microscopy (SSM) is one of the techniques which has been widely used to diagnose active PTB [53,54,55], identifying acid-fast bacilli through acid-fast stainings such as Ziehl–Neelsen (hot stain) or Kinyoun (cold stain), whose principle involves the binding of carbol fuchsin to the mycobacterial mycolic acids [6].

Ziehl–Neelsen staining is cost-effective (mean cost USD 13.31) [56] but may underestimate bacterial burden [57,58], as well as presenting variable sensitivity (32% to 89%) [56]. Furthermore, this technique cannot differentiate between dead and live bacteria, drug-susceptible from drug-resistant strains, nor distinguish *M. tuberculosis* from other mycobacteria [46], exhibiting compromised sensitivity whenever the bacterial load is less than 10,000 bacilli/mL sputum sample [59]. A study performed by Chopra et al. [60] showed that Kinyoun’s method presented higher sensitivity (98.37%) compared to Ziehl–Neelsen staining (89.25%), indicating it is more effective at detecting tubercle bacilli.

Fluorescence or light-emitting diode (LED) microscopy [61,62,63], automated microscopy, and artificial intelligence (AI) [64,65,66,67] techniques have been employed to improve the performance of SSM.

Notably, SSM should be combined with other diagnostic tools to enhance the efficacy and reliability of TB diagnosis [68]. Despite the diffused employment of molecular approaches, researchers underscore that SSM remains fundamental as the primary diagnostic technique for TB globally [69,70,71], with high-burden countries conducting millions of smears annually [72,73].

### 2.2. Chest Radiography

Chest radiography/chest X-ray (CXR) is very useful for TB diagnosis, particularly in resource-limited settings, showing high sensitivity in detecting PTB but limited specificity [74,75]. According to the World Health Organization (WHO) [76], this technique exhibits a sensitivity ranging from 87% to 98% and is considered a valuable tool for screening PTB [77]. Moreover, CXR provides diagnostic and prognostic information in children with TB [78] despite its limitations in detecting lymphadenopathy, which requires additional methods [79]. Key radiographic findings frequently observed on CXRs in patients diagnosed with PTB encompass cavitation, consolidation, masses, pleural effusion, calcification, and nodules [80], with predominant features often comprising cavitation, bronchiectasis, and fibrosis [81]. Due to the overlap of radiographic findings, another limitation of CXR is its inability to differentiate PTB from pulmonary diseases caused by NTM [82,83].

CXR as a screening test shortens the period required to diagnose PTB [84]. Furthermore, the rise of AI, particularly deep learning algorithms, has revolutionized TB detection, showing superior performance in both screening and diagnosis [85,86]. AI approaches have opened new possibilities for improved accuracy and efficiency in TB diagnosis, aligning with the WHO’s recommendation to use Computer-Aided Detections (CAD) in conjunction with human interpretation for TB screening in individuals aged 15 years or older [87].

A notable breakthrough is the deep convolutional neural network (DCNN)-based AI algorithm developed by Nijiati et al. [88], which demonstrated an impressive accuracy of 96.73% in diagnosing TB from CXR, surpassing the performance of other models. Notably, incorporating demographic variables such as age, weight, height, and gender has enhanced the performance of deep learning models [89]. Additionally, Rahman et al. [90] underscored the importance of segmenting lung regions in CXR images before applying deep learning classification. Their study revealed that the DenseNet201 model achieved a higher accuracy of 98.6% when using segmented lung images compared to 96.47% when using whole X-ray images.

Despite the advancements in CAD for TB detection, concerns persist regarding potential biases, limitations in implementation, and challenges in its broader applicability. Ongoing evaluation, consideration of version updates, and a tailored approach to implementation that considers local data and patient characteristics are crucial to ensure the effectiveness and safety of CAD software in clinical practice [91]. Challenges include limited disease detection, suboptimal accuracy for specific populations, and the need for validation for pediatric use [92].

Currently, 16 CADs for digital chest radiography are planned for WHO policy review [93], signaling the collaboration between AI and conventional diagnostic methods.

## 3. Culture-Based Diagnostic

*M. tuberculosis* is classified as a slow-growing mycobacteria, requiring seven days to six weeks to produce visible colonies on solid media. The procedure for culturing *M. tuberculosis* must be performed only in Biosafety Level 3 or 4 laboratories due to the high transmissibility of the mycobacteria, which is a limiting diagnostic capacity in many low and middle-income countries. Also, adequate training of laboratory analysts is essential for reducing pre-analytical risks associated with the entire diagnostic process [94].

The use of selective media enhances accuracy compared to non-selective media, as the most frequent sample is sputum, which may carry non-tuberculous bacteria from normal microbiota [3,95]. The Petroff method has been used for the digestion-decontamination of contaminated clinical samples before culturing on solid media. Clinical specimens are incubated with an equal volume of a 2 to 4% NaOH solution for approximately 15 min, followed by centrifugation, and then inoculated onto solid media [96]. Kudoh and Kudoh [97] proposed a simplified method for the decontamination step and subsequent culturing. The clinical samples are picked up using a sterile cotton swab, which is subsequently immersed into a 4% NaOH solution. After 2 min, the swab is inoculated onto slightly acidified solid media (such as Kudoh–Ogawa medium). This method has been evaluated and has proven to be as effective and sensitive as the Petroff decontamination method for sputum samples [98,99,100]. It has been used in developing countries [99,101,102], as well as in rural areas without adequate infrastructure for culturing mycobacteria [100]. Additionally, due to its speed, this method is favored in laboratories that handle a high volume of clinical samples [98].

Solid media that have been used to cultivate *M. tuberculosis* involve Löwenstein–Jensen (LJ), described by Ernest Löwenstein in 1931 and modified by Kai Adolf Jensen in 1932 [103], and Kudoh–Ogawa (KO), an alternative medium used to grow this bacterium [97]. Both LJ and KO are selective for *Mycobacterium* species due to the presence of malachite green (a triarylmethane dye) [104]. According to Madeira et al. [102], the growth of *M. tuberculosis* in these media presents high and similar sensibility and specificity. These researchers observed a lower contamination rate with KO medium (4.1%) compared to LJ medium (9.0%), suggesting a potentially higher risk of false-positive results with LJ due to contamination by NTM or other microorganisms. However, both media are time-consuming, which might lead to delays in the diagnosis of TB [102].

Regarding liquid media, Middlebrook is a medium that may vary in composition, resulting in different formulations, such as Middlebrook 7H9, 7H10, and 7H11, which primarily differ in the composition and concentration of organic compounds [105]. Traditionally, Middlebrook medium has been enriched with various compounds to enhance sensitivity [106]. Middlebrook 7H10, in particular, can be supplemented with oleic acid–albumin–dextrose–catalase. This medium supports faster mycobacteria growth and allows further biofilm studies [107,108].

In addressing the limitations of liquid media, Becton Dickinson (BD) introduced the Mycobacteria Growth Indicator Tube (MGIT), combining Middlebrook 7H9 broth with fluorescent compounds metabolized by *M. tuberculosis*. This method, revealed under ultraviolet (UV) light, shortened incubation time and enhanced detection rates during routine laboratory procedures [109,110]. Some studies have reported that MGIT 960 outperforms the use of LJ to detect *M. tuberculosis* [111,112,113].

Despite the time-consuming nature of culture, it remains the gold standard for diagnosing TB (including extrapulmonary infections) and monitoring TB treatment, offering advantages in identifying the pathogen and the antimicrobial susceptibility profile determination [114]. Furthermore, culturing mycobacteria is also the gold standard for differentiating between MTBC and NTM [69,115].

## 4. Molecular Diagnostic Techniques

Advances in molecular biology have driven research and development in methods for the rapid and accurate detection of MTBC and NTM and their drug resistance, providing important tools for TB control [116]. In fact, the introduction of molecular tests has significantly improved TB diagnosis, particularly for cases with negative microscopy [117]. However, the WHO emphasizes that molecular tests are not substitutes for microbiological cultures and phenotypic antimicrobial susceptibility testing [53].

### 4.1. Molecular WHO-Recommended Rapid Diagnostic Tests

WHO recommends the use of molecular rapid diagnostic tests to detect *M. tuberculosis* and resistance to rifampicin (RIF) and fluoroquinolones. These tests are used to diagnose and guide TB treatment, and can also be used to monitor the response to treatment [118].

#### 4.1.1. Nucleic-Acid Amplification Tests

Most WHO-recommended rapid diagnostic tests for TB rely on nucleic-acid amplification technology (NAAT) (Table 1) [119]. Critical examples for initial TB diagnosis without antimicrobial resistance assessment include the Loopamp™ MTBC Detection Kit and FluoroType^®^ MTB [118].

The Loopamp™ MTBC Detection Kit (TB-LAMP; Eiken Chemical, Tokyo, Japan) is a manual assay that provides results in less than one hour. The loop-mediated isothermal amplification (LAMP) technology [138] utilizes four primer sets to amplify six distinct regions within the DNA of MTBC. This process forms stem-loop structures detectable by dyes like SYBR green and calcein [120,139,140]. Nagai et al. [119] reported an approximate sensitivity of 80.9% and specificity of 96.5%. TB-LAMP holds potential as a substitute for SSM in diagnosing PTB in adults with symptoms and as a follow-up test to SSM, especially for further examination of smear-negative samples [120].

FluoroType^®^ MTB VER 1.0 and VER 2.0 (Bruker/Hain Lifescience, Nehren, Germany) utilize FluoroType^®^ technology for rapid molecular genetic testing, allowing direct detection of MTBC from patient samples. These automated systems deliver results within three hours, using fluorescence-based technology for enhanced reliability and efficiency in TB diagnostics [121]. The assay utilizes high-resolution melt analysis to identify and automatically record fluorescence signals linked to probes that target the MTBC insertion element IS*6110* [141]. It is important to highlight that a remarkable characteristic of FluoroType^®^ MTB VER 1.0 relies on its ability to detect clinically relevant NTM species [142].

Moreover, WHO has endorsed several mWRD tests for the initial TB diagnosis and the detection of resistance to RIF or the combination RIF/INH [118]. Among these, the Xpert^®^ MTB/RIF Assay (Cepheid, Sunnyvale, CA, USA) is an automated diagnostic test using nested real-time PCR (qPCR) to detect the MTBC and RIF resistance qualitatively. It amplifies a specific segment of the *rpoB* gene and distinguishes between wild-type and mutation-associated RIF resistance. The assay is compatible with GeneXpert^®^ systems and automates sample processing and nucleic acid amplification [125]. Furthermore, the Xpert^®^ MTB/RIF Ultra assay, also developed by Cepheid, is an improved version of the Xpert^®^ MTB/RIF test, offering higher sensitivity and reliability for detecting MTBC and RIF resistance. This is achieved through two multicopy amplification targets (IS*6110* and IS*1081*), a larger PCR chamber, and melting curve analysis. Xpert Ultra exhibits a lower LoD (16 CFU/mL) compared to 131 CFU/mL for Xpert^®^ MTB/RIF) [69].

Chip-based qPCR technology is employed by Truenat^®^ MTB and Truenat^®^ MTB Plus (Molbio Diagnostics, Goa, India) to detect *M. tuberculosis* in specimens from pulmonary and extrapulmonary TB [69]. Truenat^®^ MTB targets the *nrdB* gene (codes for R2-like ligand binding oxidase) with an LoD of 100 CFU/mL [128]. At the same time, Truenat^®^ MTB Plus utilizes the *nrdZ* gene and the IS*6110* sequence, offering a significantly lower LoD of 29 CFU/mL [129]. Following a positive Truenat™ MTB Plus test, an aliquot of the extracted DNA undergoes further testing with the Truenat MTB-RIF-Dx assay to detect mutations related to RIF resistance [69]. An important drawback of Truenat^®^ MTB is its inability to detect mycobacterial species other than *M. tuberculosis* [128].

The RealTime MTB assay (Abbott Molecular, Des Plaines, IL, USA) uses PCR to detect MTBC DNA, exhibiting a sensitivity of 93% in culture-positive samples, 81% in smear-negative culture-positive samples, and a specificity of 97%. With an LoD of 17 CFU/mL, the target regions include IS*6110* and *pab* gene (codes for protein antigen B, Pab) [130]. The RealTime MTB RIF/INH (Abbott Molecular, Des Plaines, IL, USA) assay detects resistance to RIF (*rpoB* gene) and different levels of INH resistance (*katG* gene and *inhA* promoter region) [132]. It allows for the identification of MTBC with or without rifampicin-resistant TB (RR-TB), isoniazid-resistant rifampicin-susceptible TB (Hr-TB), or multidrug-resistant TB (MDR-TB) within 10.5 h using raw or processed sputum specimens or DNA eluates from positive samples [69].

The BD MAX™ MDR-TB assay (Becton Dickinson, Sparks, MD, USA) integrates MTBC detection and assesses resistance to RIF and INH. The automated process involves specimen treatment with BD MAX STR reagent, transfer to the BD MAX MDR-TB Sample Tube, and utilization of the BD MAX System for DNA extraction, amplification, and detection using qPCR. Targeting IS*6110* and IS*1081* for MTBC detection (LoD = 0.5 CFU/mL; 92.6% overall sensitivity, and 98.6% overall specificity), the system also identifies resistance, with the *rpoB* gene for RIF resistance, and the *inhA* promoter region and *katG* gene for INH resistance [133].

The FluoroType^®^ MTBDR VER 2.0 (Bruker/Hain Lifescience, Nehren, Germany) is a multiplex qPCR assay for the rapid detection of MTBC and resistance to RIF and INH, based on the LiquidArray^®^ technology. DNA extraction can be manual (FluoroLyse) or automated (GenoXtract^®^ X2 cartridge). The targeted sequences include *rpoB*, *katG*, and *inhA*, allowing the identification of mutations associated with RIF and INH resistances [134].

The cobas^®^ MTB test (Roche Molecular Diagnostics, Pleasanton, CA, USA) targets *16S rRNA* and *esx* genes through qPCR (LoD = 8.8 CFU/mL in raw sputum). It is intended for use on the cobas^®^ 5800/6800/8800 Systems and is an automated, qualitative in vitro diagnostic test. The test can detect MTBC DNA in various human respiratory specimens, including sputum and bronchoalveolar lavage samples [135]. Additionally, the cobas^®^ MTB-RIF/INH test (Roche Molecular Diagnostics, Pleasanton, CA, USA), also conducted on the cobas^®^ 5800/6800/8800 Systems, is an automated PCR test designed as a reflex test with cobas^®^ MTB. It identifies RIF and INH resistance mutations in *M. tuberculosis*, analyzing eighteen RIF-resistance-associated mutations in the *rpoB* gene and seven INH-resistance-associated mutations in the *katG* gene and *inhA* gene promotor region. The LoD in raw sputum is 182 CFU/mL for RIF-resistant *M. tuberculosis* and 27.5 CFU/mL for INH-resistant *M. tuberculosis* [137].

The aforementioned Abbott RealTime MTB and Abbott RealTime MTB RIF/INH, BD MAX™ MDR-TB, FluoroType^®^ MTBDR and FluoroType^®^ MTB, and cobas^®^ MTB and cobas MTB-RIF/INH have been classified as Moderate complexity automated (MC-a) NAATs for detection of TB and resistance to RIF and INH [69]. Overall, WHO prioritizes using mWRDs, including MC-aNAATs, as the initial diagnostic test for suspected TB. The use of molecular approaches, in addition to culture methods, offers several advantages, including faster results, improved sensitivity, rifampicin resistance detection, and, ultimately, quicker initiation of appropriate treatment [69]. All the NAATs presented in Table 1 (except for Truenat^®^ MTB) detect DNA from MTBC but do not differentiate the subspecies, especially *Mycobacterium bovis*, which is a significant cause of zoonotic TB. This is particularly important as the zoonotic transmission of TB, especially that involving *M. bovis*, has gained increasing attention globally [143,144,145]. Therefore, because these methods do not differentiate between these two species, crucial information about the zoonotic aspect of TB may be overlooked, and a traditional culture-based method must be performed [125,128,129].

#### 4.1.2. Line Probe Assays (LPAs)

Line-probe assays (LPAs) represent a category of DNA strip-based tests that detect the presence of MTBC and mutations associated with drug resistance. The interpretation of the results involves analyzing a band pattern on the strips, where immobilized probes are bound to MTBC amplicons. The probes are specifically designed to target prevalent mutations linked to resistance against both first- and second-line anti-TB drugs, as well as distinct MTBC wild-type DNA sequences. Two commonly used LPAs, GenoType^®^ MTBDRplus and GenoType^®^ MTBDRsl, are recommended by the WHO [146].

GenoType^®^ MTBDRplus (VER 2.0; Hain Lifescience GmbH, Nehren, Germany) enables the identification of RIF resistance by detecting significant mutations in the *rpoB* gene. Moreover, it assesses INH resistance through the *katG* gene and the promoter region of the *inhA* gene. Benefits include testing from patient specimens or cultures and obtaining results in five hours (compared to 1 to 2 months required by conventional methods) [147]. In a study conducted by Moga et al. [148], GenoType^®^ MTBDRplus VER 2.0 achieved a sensitivity of ~94.3% to detect INH resistance among MDR-TB isolates and a specificity of 100%. Similarly, Stephen et al. [149] reported 100% sensitivity and specificity to detect resistance to both INH and RIF. Furthermore, Tan et al. [150] reported that the test displayed a sensitivity of ~92.7% and a specificity of ~94.5% in diagnosing TB, while Meaza et al. [151] reported a sensitivity and specificity of ~96.4% and 100%, respectively, to detect MDR-TB.

GenoType^®^ MTBDRsl (VER 1.0 and VER 2.0; Hain Lifescience GmbH, Nehren, Germany) assists in detecting extensively drug-resistant tuberculosis (XDR-TB) by identifying MTBC and its resistance to fluoroquinolones, aminoglycosides/cyclic peptides, second-line injectable drugs (KAN: kanamycin; AMK: amikacin; CAP: capreomycin) and ethambutol (only VER 1.0) [152]. According to Bouzouita et al. [153], GenoType^®^ MTBDRsl 2.0 presented sensitivities of approximately 92.8% and 80% to detect resistance to fluoroquinolones and second-line injectable drugs (KAN, AMK, and CAP), respectively. Notably, specificity was 100% for the drugs mentioned above.

Detecting MDR-TB is a notable strength of LPAs. Given that MDR-TB poses a substantial public health risk, timely identification is essential for effectively treating and preventing the spread of the disease [154]. Another advantage is the ability of LPAs to detect specific genetic mutations associated with drug resistance. This allows for personalized treatment plans, as different *M. tuberculosis* strains may respond differently to different drugs [146].

However, drawbacks include the potential for false results, reliance on skilled personnel, and higher costs compared to traditional methods. Despite challenges, line probe assays are crucial in managing MDR-TB due to their ability to identify drug resistance-associated genetic markers [146].

### 4.2. Sequencing

Several studies highlight the impact of sequencing on diagnosing infectious diseases [155,156,157]. In 2018, WHO published one of the first guides concerning the use of whole genome sequencing (WGS) as a tool to study MTBC, particularly the mutations related to drug resistance. The document discussed the use of the sequencing platforms Illumina MiSeqTM (Illumina Inc., San Diego, CA, USA), Ion Personal Genome Machine^®^ (Thermo Fisher Scientific, Inc., Waltham, MA, USA), Nanopore MinION^®^ (Oxford Nanopore Technologies, Oxford, UK), and the GeneReader system (Qiagen, Hilden, Germany) [158]. Similarly, in October 2023, WHO published a document to help laboratories implement next-generation sequencing (NGS), also known as high throughput sequencing (HTS), for TB bacteria characterization, focusing on drug resistance mutations to complement existing TB surveillance systems [159]. The study by Vogel et al. [160] demonstrated the technical and financial aspects of implementing WGS in a National Reference Laboratory in Kyrgyzstan. The authors highlighted that implementing WGS for TB diagnostics involves challenges such as higher sequencing costs, extended procurement and capacity building timelines, early consideration of infrastructure requirements, tailored solutions for quality assurance, careful planning for transitioning WGS to routine diagnostics, and the necessity of ongoing support by experienced experts for sustainable success. A review by Ness, DiNardo, and Farhat [161] presents sequencing platforms for NGS of *M. tuberculosis*, as well as applications of targeted HTS in the context of TB.

### 4.3. MALDI-TOF MS

Regarding mass spectrometry, Matrix-Assisted Laser Desorption/Ionization Time-of-Flight Mass Spectrometry (MALDI-TOF MS) is revolutionizing clinical microbiology by rapidly and accurately identifying microorganisms in various samples, potentially improving patient outcomes, and reducing hospital stays [162]. Alcolea-Medina et al. [163] successfully established a fast and affordable method (MALDI-TOF) for identifying mycobacteria species in hospitals. It achieved 85% accuracy compared to existing methods, making it an attractive tool for clinical use.

Some studies present the use of nucleotide MALDI-TOF-MS and MALDI-TOF-MS as promising rapid tools for detecting drug resistance in *M. tuberculosis* [164,165,166], as well as the simultaneous detection of MTBC and mutations related to drug resistance [167,168,169], although these techniques cannot identify de novo drug resistance mutations [164] nor detect mutations related to novel resistance mechanisms [166].

MALDI-TOF MS is a rapid and cost-effective method for microbial identification. While molecular-based assays like PCR offer high specificity and sensitivity, they are generally more expensive and time-consuming. In laboratories equipped with MALDI-TOF MS, it is often the preferred method due to its ease of use and speed [163].

Although a pure culture enhances the specificity and sensitivity of the technique, some studies have shown that MALDI-TOF MS can be performed on raw specimens such as sputum and bronchoalveolar lavage fluid [167,168,170].

### 4.4. Biosensors

Lastly, biosensors are versatile biomedical diagnostic tools using targeted molecules to detect analytes [171] that usually consist of a biological sensing element along with a physicochemical transducer and a processor [172]. Notably, in TB diagnosis, DNA electrochemical biosensors, usually targeting the IS*6110*, demonstrate exceptional sensitivity, holding potential for drug-resistance identification [173,174]. In addition to DNA, biosensors can target antigens such as CFP-10/ESAT-6, MPT64, and Ag85, as well as antibodies, and cytokines [172]. Furthermore, biosensors are categorized based on their sensing technologies. In addition to electrochemical biosensors, there are other types, such as surface plasmon resonance (SPR), optical, mechanical, and quartz crystal microbalance (QCM)-based biosensors [175].

## 5. Immunological Approaches

The interaction between *M. tuberculosis* and the host involves a complex interplay of immune responses, influencing infection outcomes [176]. In TB diagnosis, immunology relies on important tools to detect adaptive immune responses in humans, such as interferon-gamma release assays (IGRAs) and tuberculin skin tests (TST), which are recommended by WHO for detecting LTBI [177]. However, no gold-standard technique exists for diagnosing this form of infection, and neither TST nor IGRAs are entirely accurate in predicting disease risk. Positive results from these tests should be considered alongside other clinical factors [178].

A comprehensive review by McIntyre et al. [179] highlighted that no serological tests have yet met WHO criteria for TB diagnosis, underscoring a gap in the understanding of the role of antibodies in TB immunity. Despite ongoing research, this incomplete knowledge complicates the development of effective vaccines, diagnostic tools, and treatments. Recent advances include the introduction of novel *M. tuberculosis* antigen-based skin tests (TBSTs) since 2022, which aim to enhance diagnostic accuracy alongside IGRAs and TSTs [180]. Additionally, a review by Melkie et al. [181] concluded that antibody tests remain insufficiently reliable for routine TB diagnosis, underscoring the need for further research and development in this area.

### 5.1. Interferon-Gamma Release Assays (IGRAs)

Interferon-gamma release assays (IGRAs) are blood-based tests that measure the production of interferon-gamma (IFN-γ) in response to *M. tuberculosis* antigens [182]. WHO recommends both IGRAs and TST for detecting LTBI [182], but IGRAs offer several advantages over TST, including a single-visit requirement and no influence by prior Bacille Calmette–Guérin (BCG) vaccination [183].

Despite their advantages, IGRAs are more expensive and require specialized laboratory equipment, which may not be readily available in low- and middle-income countries [183]. Furthermore, IGRAs yield indeterminate results in approximately 1 in 26 tests, particularly for immunocompromised individuals and young children [184].

Currently, three WHO-recommended IGRAs include:

- T-SPOT^®^.TB (T-Spot; Oxford Immunotec Ltd., Oxford, UK): uses the enzyme-linked immunospot (ELISPOT) method to count *M. tuberculosis*-sensitized T cells [185];
- QuantiFERON^®^-TB Gold Plus (QFT-Plus; Qiagen, Hilden, Germany): a fourth-generation assay that measures the cell-mediated immune response to two specific *M. tuberculosis* antigens—Early Secreted Antigenic Target 6 (ESAT-6) and Culture Filtrate Protein 10 (CFP-10)—using an ELISA-based approach [186];
- WANTAI TB-IGRA (Beijing Wantai Biological Pharmacy Enterprise Co Ltd., Beijing, China): an ELISA-based IGRA test similar to QFT-Plus, using a recombinant fusion protein of CFP-10 and ESAT-6 antigens [187].

Despite not being usually recommended for the diagnosis of active TB [156], several studies have explored the potential of IGRAs for this purpose [188,189,190,191,192].

Three new IGRAs—(1) Advansure TB IGRA (LG Chem, Seoul, Republic of Korea), (2) Lioferon TB/LTBI (LIONEX Diagnostics & Therapeutics GmbH, Braunschweig, Germany), and (3) Quantiferon-Diasorin (Stillwater, MN, USA)—are under review for potential WHO policy recommendations [93].

### 5.2. Tuberculin Skin Test (TST)

Tuberculin skin testing (TST), also known as the Mantoux test, involves the intradermal injection of 0.1 mL of tuberculin-purified protein derivative (PPD) into the forearm, creating a 6 to 10 mm wheal [193]. This wheal typically disappears within 15 to 20 min as the liquid is absorbed. The test is evaluated by a healthcare worker 48 to 72 h later by measuring the diameter of the induration (the raised, hardened area) in millimeters, which results from a delayed hypersensitivity reaction. The measurement excludes any surrounding erythema (redness) [194]. A result is considered positive for individuals devoid of risk factors for TB if the induration measures ≥15 mm; however, in immunocompromised patients or those receiving immunosuppressive treatments, a cut-off of ≥5 mm is used [194]. BCG vaccination significantly impacts TST specificity [183]. Nevertheless, BCG history does not interfere with TST results in children over three years old. For those under three, for whom BCG may cause false positives, using IGRAs is recommended. In situations in which IGRAs are unavailable or inconclusive, ignoring the BCG vaccination history is advised [195].

Despite limitations, TST remains a valuable tool for LTBI detection due to its cost-effectiveness and field applicability. However, it requires a cold chain for PPD storage and transportation, two healthcare visits, and specific training in intradermal injection, reading, and interpretation [183]. Although PPD requires a cold chain for storage and transportation [183], a study developed by Maes et al. [196] shows that tuberculin is not as heat labile as commonly believed. This finding is crucial since it suggests that tuberculin may be more resilient to temperature fluctuations than previously thought, emphasizing the importance of proper handling while offering reassurance regarding its viability even if cold chain is compromised. In general, TST continues to be clinically significant in both low and high TB-endemic regions until more advanced and widely available tests become accessible [197].

### 5.3. Mycobacterium Tuberculosis Antigen-Based Skin Tests (TBSTs)

Emerging as alternatives to traditional TST, TBSTs like Cy-Tb™ (Serum Institute of India, Pune, India), Diaskintest^®^ (Generium, Moscow, Russian Federation) and C-TST (Anhui Zhifei Longcom, Chongqing, China) offer improved specificity and sensitivity for TB diagnosis [180]. The tests involve intradermal injections with *M. tuberculosis* antigen-based reagents and assess induration 48–72 h later. Cy-Tb™ employs a unique test dose (0.1 mL containing 0.05 μg each of recombinant dimer ESAT-6 (rdESAT-6) and recombinant CFP-10 (rCFP-10), boasting high accuracy (73.9% sensitivity, 99.3% specificity) [180,198]. Diaskintest^®^ utilizes recombinant proteins (CFP-10 and ESAT-6), with hyperallergic reactions like blistering and necrosis considered rare [180], achieving approximate sensitivity and specificity of 91.18% and 99.15%, respectively [199]. Moreover, C-TST, formerly EC-Test, uses a similar antigen and assessment method [180], reaching 90.6% and 88.2% of sensitivity and specificity, respectively [193]. These TBSTs represent a promising advancement in TB diagnosis, offering improved accuracy and potentially reducing limitations associated with the traditional TST.

Regarding safety, a systematic review by Hamada et al. [200] found that these TBSTs showed safety profiles similar to TSTs, with mainly mild injection site reactions. This suggests their potential as alternatives, especially considering their accuracy near IGRA tests.

Despite their ease of administration and favorable safety profile, novel TBSTs have not yet surpassed the use of TST and IGRAs in clinical practice [200,201]. However, further data regarding TBSTs are needed, particularly for pregnant women [200].

## 6. Ongoing Research

Recent studies showcase promising advancements in TB diagnostics:

1. Cepheid MTB-HR cartridge: This fingerstick blood test identifies a three-gene transcriptomic signature, achieving a sensitivity of 59.8% in distinguishing TB from non-TB cases. Combined with other methods, it identified 71.2% of confirmed TB cases [202].
2. Immuno-affinity LC-MS (ILM) assay: This novel approach quantifies peptides from HIV-1 and *M. tuberculosis* proteins, achieving high sensitivity and specificity for both infections [203]. Additionally, it can differentiate treatment responders from non-responders, providing valuable insights for integrated TB and HIV management [203].
3. CAPTURE-XT technology: This “lab-on-a-chip” platform uses dielectrophoresis to isolate *M. tuberculosis* from sputum, enabling efficient bacterial purification for subsequent molecular confirmation. It demonstrated high concordance with culture diagnosis, highlighting its potential as a robust sample preparation tool [204].
4. Electronic nose (EN): Ketchanji Mougang et al. [205] conducted a study in Douala, Cameroon, assessing an EN for diagnosing PTB in a clinical setting. The EN utilizes eleven quartz microbalance sensors modified with metalloporphyrins and corroles to detect volatile organic compounds (VOCs) present in exhaled breath samples collected using a specialized breath sampler. Breath samples were segregated into alveolar and non-alveolar fractions, with analysis focusing exclusively on the alveolar portion to minimize external contaminants. The sensors detect changes in frequency resulting from interactions with VOCs, which exhibit unique patterns associated with TB. The EN demonstrated an accuracy of 88.0%, with a sensitivity of 90.8% and specificity of 85.7%, effectively distinguishing between PTB patients and healthy controls. Notably, the sensitivity of the EN was comparable to TB-LAMP and CXR, surpassing SSM.

These studies offer hope for improved TB diagnosis, particularly in challenging settings. Further research and development are crucial to translate these technologies into practical applications, contributing to global efforts for TB elimination.

### Tests Undergoing WHO Policy Review

Various innovative diagnostic tools for TB have emerged, and several of these promising technologies are currently undergoing rigorous evaluation by the WHO to assess their suitability for incorporation into global TB diagnostic guidelines [93].

Concerning the culture-based drug susceptibility testing, Sensititre™ *Mycobacterium tuberculosis* MYCOTBI AST Plate (Thermo Fisher Scientific, Inc., Waltham, MA, USA) is a manual semiquantitative test whose results can be interpreted visually or with the aid of the ThermoScientific™ Sensititre™ Vizion™ System [206]. The test is based on microbroth dilution, testing 12 drugs: the first-line antibiotics rifampicin, rifabutin, isoniazid, and ethambutol, as well as the second-line antibiotics ofloxacin, moxifloxacin, amikacin, kanamycin, streptomycin, para-aminosalicylic acid, ethionamide, and cycloserine. Minimum inhibitory concentration (MIC) results can be obtained from 7 to 21 days [206].

Fujifilm SILVAMP TB LAM test (FujiLAM; Fujifilm, Tokyo, Japan), like the Abbott LF-LAM, detects lipoarabinomannan in urine samples [207], with 70% and 93% of sensitivity and specificity to detect TB in adults, and 51% and 87% for children [208]. This new point-of-care test has been considered suitable to detect PTB as well as extrapulmonary forms of the disease in patients with HIV [209] and is easy to be performed by any healthcare worker [210].

The three IGRAs currently undergoing WHO policy review involve (1) StandardTM E TB-Feron ELISA (SD Biosensor, Gyeonggi-do, Republic of Korea), (2) STANDARDTM F TB-Feron FIA (SD Biosensor, Gyeonggi-do, Republic of Korea), and (3) VIDAS^®^ TB-IGRA (bioMérieux, France) [93]. The first is not influenced by previous BCG vaccination; results are available in approximately 100 min and present 98.03% and 98.55% sensitivity and specificity, respectively [211]. Concerning the efficacy of StandardTM E TB-Feron ELISA compared to QFT-Plus, accordance of 92% between the tests has been reported by Yoo et al. [212] and 94% by Jung et al. [213]. STANDARDTM F TB-Feron FIA, in turn, quantifies the IFN-γ in the blood samples through a fluorescent immunoassay (FIA) technique, delivering results within 15 min [214]. In South America, Saint-Pierre et al. [215] reported a sensitivity of 88.59% and a specificity of 92.5% for this test. Lastly, VIDAS^®^ TB-IGRA exhibits sensitivity and specificity of 97.5% and 97.6%, respectively, with results available within 17 h (estimative for one patient) [216]. Petruccioli et al. [217] reported that the test is able to detect the IFN-γ response in CD4^+^/CD8^+^ T-cells for both TB and LTBI. Diagbouga et al. [218] also discussed that the test is promising for both active and latent TB.

Low complexity automated NAATs undergoing WHO policy review include the all-in-one cartridges STANDARD™ M10 MDR-TB (SD Biosensor, Gyeonggi-do, Republic of Korea) and IRON-qPCR™ RFIA Kit (Bioneer, Daejeon, Republic of Korea) [93]. STANDARD™ M10 MDR-TB performs the simultaneous detection of *M. tuberculosis* and resistance to RIF and INH from sputum samples based on qPCR technology, with a turnaround time of 80 min [219]. IRON-qPCR™ RFIA Kit is undergoing a clinical trial estimated to be completed in 2024. The test detects *M. tuberculosis* and mutations related to RIF, INH, fluoroquinolones, and aminoglycosides resistance, which is relevant since there are no WHO-endorsed tests to detect resistance to first- and second-line drugs to treat TB [220].

Finally, the DeepChek^®^ Assay 13-Plex KB Drug Susceptibility Testing (ABL SA, Luxembourg, Luxembourg) assesses TB drug resistance through NGS. The test detects resistance-associated mutations in specific *M. tuberculosis*-targeted genes by sequencing. The key steps involve DNA extraction, multiplex PCR, NGS, and data analysis [221].

## 7. Final Remarks

**Figure 1:**
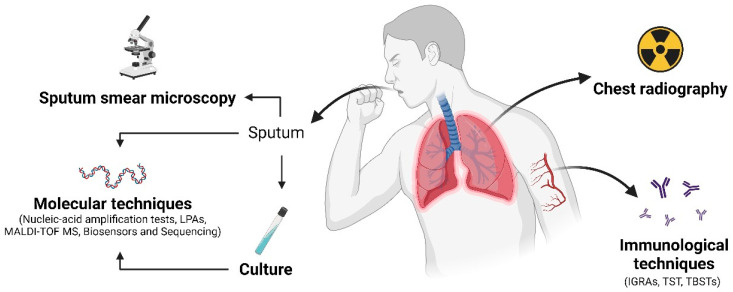
This figure illustrates relevant diagnostic techniques employed for pulmonary tuberculosis diagnosis. Sputum is collected and examined under a microscope for acid-fast bacilli (AFB). Chest radiography assesses lung abnormalities such as infiltrates, cavities, or pleural effusions. Molecular techniques detect the genetic material of MTBC using methods like NAATs, LAMP, MALDI-TOF MS, biosensors, and sequencing. Immunological tests, such as IGRAs, TST, and TBSTs, measure the body’s immunity in response to mycobacterial proteins. Culture involves growing the mycobacteria in a laboratory for identification and further antimicrobial susceptibility testing. These techniques collectively aid in the diagnosis of pulmonary tuberculosis. Created with BioRender.com.

In order to summarize the methods discussed in this review, an overview of the advantages and drawbacks of these methods, as well as the main diagnostics approaches used to diagnose pulmonary tuberculosis, can be visualized in Table 2 and Figure 1, respectively.

Finally, although the diagnosis of extrapulmonary TB is not the focus of this review, it is important to highlight the clinical relevance of the assessment of adenosine deaminase (ADA), which has been extensively used to diagnose these forms of tuberculosis [222,223,224]. ADA, an enzyme found in some types of leukocytes and crucial for purine metabolism, is associated with intracellular infections [225]. Elevated pleural fluid ADA levels are a useful marker for diagnosing tuberculous pleurisy (TPE), especially in high TB burden areas, though they can also be high in other conditions. Low ADA levels can help exclude TPE, prompting further investigation to identify the cause of pleural effusion [224]. ADA is vital for regulating immune, neurological, and vascular functions and aids in lymphocyte development. Elevated serum ADA can indicate various conditions that stimulate the immune system, including TB. However, ADA levels alone are not reliable for differentiating PTB from other lung infections [226].

## 8. Conclusions

Tuberculosis remains an important cause of death among infectious diseases, with granulomas as the hallmark of its pathophysiology. Since a wide range of the population is estimated to be infected with *M. tuberculosis*, exhibiting no symptoms, the infection can become active upon a series of factors, including the interaction between the pathogen and the host immune system. An important consequence of tuberculosis reactivation is the significant risk of transmitting *M. tuberculosis* to other individuals, which can amplify the spread of the disease within the community.

This review addresses the multiple approaches to diagnosing tuberculosis, focusing on pulmonary tuberculosis. Despite the availability of several molecular testing techniques, they are not accessible in various settings, especially in low- and middle-income countries. Here, culture-based methods play a critical role. The culture of *M. tuberculosis* not only remains the gold standard for diagnosis but also allows for the characterization of the pathogen, including drug susceptibility profile. This is crucial for ensuring effective treatment regimens and controlling the spread of drug-resistant strains. In many resource-limited settings, culture-based methods offer a vital approach to diagnosis where advanced molecular tests may not be feasible.

When feasible, the scenario of tuberculosis diagnosis can be improved with molecular testing without neglecting culture-based methods and SSM, thus improving specific identification of the etiological agent and the drug susceptibility testing, along with the use of decentralized and multi-disease testing (especially *M. tuberculosis*/HIV coinfection). Importantly, the tests should be affordable and favor non-sputum samples, such as oral swabs and urine. These samples are preferable since sputum samples are more difficult to obtain, making them a critical consideration for improving accessibility and convenience in testing [227].

## Figures and Tables

**Table 1:** Nucleic acid amplification tests (NAATs) recommended by the World Health Organization for tuberculosis diagnosis.

Test	Target	LoD CFU/mL	Sensitivity (%)	Specificity (%)	Time to Result (h)	RIF/INH Resistance Detection	Notes	References
Loopamp^TM^ MTBC	6 regions within MTBC DNA	N/A	~80.9	~96.5	<1 h	No	Manual assay	[119,120]
FluoroType^®^ MTB VER 1.0	IS*6110*	30	~88.1	~98.9	3 h	No	Uses FluoroType^®^ technology, manual/automated DNA extraction	[121,122,123]
FluoroType^®^ MTB VER 2.0	IS*6110*	2.6	~91.6	~93.8	3 h	No	Uses FluoroType^®^ technology, manual DNA extraction	[121,124]
Xpert^®^ MTB/RIF	*rpoB*	131	~85	~98	2 h	RIF	Automated, nested qPCR	[69,125,126]
Xpert^®^ MTB/RIF Ultra	IS*6110*, IS*1081*, *rpoB*	16	~87.8	~98.1	<2 h	RIF	Improved version of Xpert^®^ MTB/RIF	[69,127]
Truenat^®^ MTB	*nrdB*	100	~73.3	~97.9	<1 h	No	Chip-based qPCR	[128]
Truenat^®^ MTB Plus	*nrdZ*, IS*6110*	29	~91.7	~97.2	<1 h	No	Chip-based qPCR, higher sensitivity than Truenat^®^ MTB	[129]
RealTime MTB	IS*6110*, *pab*	17	93% (culture+), 81% (smear-negative)	97%	~6 h	No	Separate test available for RIF/INH resistance	[130,131]
RealTime MTB RIF/INH	*rpoB*, *inhA* promoter, *katG*	60	94.8 (RIF) 88.3 (INH)	100 (RIF) 94.3 (INH)	Additional 4.5 h	RIF/INH	Carried out upon positive RealTime MTB result	[69,132]
BD MAX™ MDR-TB	IS*6110*, IS*1081*, *rpoB*, *inhA* promoter, *katG*	0.5	92.6	98.6	<4 h	RIF/INH	Integrated MTBC and RIF/INH detection	[133]
FluoroType^®^ MTBDR VER 2.0	*rpoB*, *katG*, *inhA*	20	89.8	97.5	2.5 h	RIF/INH	Integrated MTBC and RIF/INH detection	[122,124,134]
cobas^®^ MTB	16S rRNA, *esx* genes	8.8	~93.5	~98.	3.5 h	No	Different targets compared to other tests	[135,136]
cobas^®^ MTB-RIF/INH	*rpoB*, *katG*, *inhA* promoter	182 (RIF) 27.5 (INH)	~96.7 (RIF) ~97.4 (INH)	~97.9 (RIF) ~99.3 (INH)	Additional 3.5 h	RIF/INH	Carried out upon cobas^®^ MTB positive result	[136,137]

LoD: limit of detection; RIF: rifampicin; INH: isoniazid; MTBC: *Mycobacterium tuberculosis* complex; N/A: not available; SSM: sputum smear microscopy; CFU: colony-forming unit; qPCR: real-time PCR.

**Table 2:** Overall advantages and disadvantages of the tests currently used to diagnose tuberculosis addressed in this review.

Technique	Advantages	Disadvantages	References
Sputum smear microscopy (SSM)	Rapid, cost-effective, important primary diagnostic technique	Low sensitivity, cannot differentiate between live and dead bacteria, cannot differentiate between Mtb and other mycobacteria	[46,53,54,55,56,69,70,71,72,73]
Chest radiography (CXR)	Cost-effective, shortens the period required to diagnose PTB, high sensitivity, can be enhanced by AI	Cannot differentiate between PTB and pulmonary infections caused by NTM	[74,75,76,82,83,84,85,86]
Culture-based methods	Gold standard, identifies the pathogen, differentiates between MTBC and NTM, improved sensitivity with liquid media	Time-consuming, requires biosafety level 3 or 4 laboratories, high cost	[3,69,94,107,108,114,115]
Nucleic acid amplification tests (NAATs)	Faster results compared to traditional methods, improved sensitivity, certain NAATs can detect antibiotic resistance, many NAATs are automated, lower LoD	Most NAATs cannot differentiate between MTBC and NTM, high cost, complexity	[69,118,119,120,121,125,128,129]
Line-probe assays (LPAs)	Rapid results, simultaneous detection of MTBC and drug resistance, high sensitivity and specificity, identify specific genetic mutations	Potential for false results, reliance on skilled personnel, high cost	[146,147,148,149,150,151,152,153]
Sequencing	Comprehensive information, improved accuracy, enhanced surveillance, personalized treatment, detection of drug resistance	High costs, technical challenges, time consuming, complex infrastructure requirements	[155,156,157,158,160]
MALDI-TOF MS	Rapid and accurate identification, cost-effective, ease of use, potential for detecting drug resistance	Lower specificity and sensitivity than PCR-based methods, requires a pure culture for optimal results	[162,163,164,165,166,167,168,169]
Biosensors	Versatility, high sensitivity, potential for drug resistance detection, point-of-care testing	Limited commercial availability, potential for interference, need for further development	[172,174]
Interferon-gamma release assays (IGRAs)	Single visit, no BCG vaccination interference, high sensitivity for LTBI progression	More expensive, requires specialized equipment, indeterminate results possible	[182,183,184]
Tuberculin skin test (TST)	Cost-effective, field-applicable	Requires two visits, cold chain for PPD, BCG vaccination affects specificity	[183]
*Mycobacterium tuberculosis* antigen-based skin tests	Improved specificity and sensitivity compared to TST, potentially reduce TST limitations	Limited safety data for pregnant women	[182,193,199]

Mtb: *Mycobacterium tuberculosis*; PTB: pulmonary tuberculosis; AI: artificial intelligence; NTM: non-tuberculous mycobacteria; MTBC: *Mycobacterium tuberculosis* complex; LoD: limit of detection; PCR: polymerase chain reaction; BCG: Bacille Calmette–Guérin; LTBI: latent tuberculosis infection; PPD: purified protein derivative.

## Supplementary Materials

The following supporting information can be downloaded at: https://www.mdpi.com/article/10.3390/diseases12090202/s1, Table S1: Some examples of tests developed for the diagnosis of non-tuberculous mycobacteria (NTM).
