# Supplementary material for: Tuberculosis Diagnosis: Current, Ongoing, and Future Approaches
Source: Diseases. 2024 Sep 3;12(9):202. doi: 10.3390/diseases12090202 (PMC11430992; doi:10.3390/diseases12090202)
Supplement: Supplementary file 1 [file diseases-12-00202-s001.zip › diseases-3105720-supplementary.pdf]

Supplementary Table S1: Some examples of tests developed for the diagnosis of non-tuberculous mycobacteria (NTM).

**Test**	**Target**	**Bacteria**	**Notes**	**Reference**
BioChip	*16S rRNA*	*M. tuberculosis,* *M. intracellulare, M. avium, M. gordonae, M. kansasii, M. fortuitum, M. scrofulaceum, M. gilvum, M. terrae, M. chelonae/M. abscessus, M. phlei, M. nonchromogenicum, M. marinum/M. ulcerans, M. aurum, M. szulgai-M. malmoense, M. xenopi, and M. smegmatis*	Microarray. 100% concordant results were observed between the BioChip and amplicons sequencing approaches. The test can be used directly in clinical specimens and after culturing.	[31]
Capilia™ TB-Neo (TAUNS Laboratories, Numazu, Japan)	MPB64	*M. tuberculosis* NTM	Immunochromatographic-based assay using immobilized monoclonal antibody anti-MPB64 onto nitrocelulose membrane. The test was performed after mycobacteria culturing in broth or solid media. NTM tested: *M. avium, M. chelonae, M. fortuitum, M. gordonae, M. intracelullare, M. kansasii, M. peregrinum*.	[32]
One-Step multiplex PCR	*rv0577* - MTBC RD750 – *M. tuberculosis* IS*1311* – *M. avium* DT1 – *M. intracellulare* *mass_3210* – *M. abscessus, M. massiliense* *mkan_rs12360* – *M. kansasii*	MTBC, *M. tuberculosis,* *M. avium, M. intracellulare mass_3210 – M. abscessus, M. massiliense mkan_rs12360 – M. kansasii*	*rv0577* and RD750 discriminate between the MTBC and NTM. The test was validated with artificial sputum spiked with *Mycobacterium* species and from clinical samples after culturing in broth media.	[33]

Table S1. *Cont.*

Test	Target	Bacteria	Notes	Reference
Duplex PCR assay	*hsp65* gene<br>Rv1458c, a putative ABC drug transporter specific for the MTBC	*M. tuberculosis*<br>NTM	Rv1458c can be used as a diagnostic marker of *M. tuberculosis*<br>NTM tested: *M. avium, M. abscessus, M. flavescens, M. fortuitum, M. intracellulare, M. kansasii, M. mucogenicum*	[34]
HRM curve analysis	*hsp65* gene	*M. tuberculosis*<br>NTM	The test was capable of identifying the following NTM: *M. abscessus, M. avium, M. fortuitum, M. gordonae, M. intracellulare, M. kansasii*	[35]
LC-MS/MS	Antigen 85B (Ag85B)	*M. tuberculosis*<br>*M. avium, M. intracellulare, M. kansasii*	The test can be used directly in clinical specimens and after culturing in broth media.	[36]
Sandwich antibody-based biosensor	Ag85B	*M. tuberculosis H37Rv, M. tuberculosis H37Ra*<br>*M. bovis BCG, M. avium, M. intracellulare, M. kansasii, M. smegmatis*	The test is based on immobilization of biotinylated monoclonal antibodies (species-specific) onto streptavidin biosensor chips, and a secondary monoclonal antibody. The antigen-antibody binding signals are detected by BLItz bio-layer interferometry biosensor. The test was validated using Middlebrook 7H9 broth culture supernatants.	[37]
BiDz-NTM$_{ST}$	Hypervariable region of *16S rRNA*	*M. tuberculosis*<br>*M. abscessus, M. avium, M. fortuitum, M. gordonae, M. intracellulare, M. kansasii*	The test is based on the use of a binary deoxyribozyme sensor that in the presence of a complementary analyte (generated by PCR) form an active deoxyribozyme catalytic core. The enzyme catalyzes the cleavage of a reporter substrate, resulting in fluorescent or colorimetric signal. The test was validated with DNA extracted after mycobacteria culturing.	[38]

Table S1. *Cont.*

Test	Target	Bacteria	Notes	Reference
GENEDIA MTB/NTM (Green Cross Medical Science Corp., Chungbuk, Korea)	IS*6110* – MTB<br>Internal transcribed spacer region and *rpoB* gene - NTM	*M. tuberculosis*<br>*M. abscessus*<br>*M. avium*	Multiplex real-time PCR. The test was performed using sputum. The analytical LoDs were 1 copy/μL, 27 copies/μL, and 953 copies/μL for *M. tuberculosis*, *M. abscessus*, and *M. avium*, respectively.	[39]
CRISPR/Cas12a	*rpoB* gene – *M. abscessus* subsp. *abscessus* and *M. abscessus* subsp. *bolletii*<br>*erm* gene – *M. abscessus* subsp. *massiliense*	*M. abscessus* subsp. *abscessus, M. abscessus* subsp. *bolletii, M. abscessus* subsp. *massiliense*	sgRNA probes are designed to target *rpoB* and *erm* genes. The target DNA is previously amplified and then mixed with CRISPR/Cas12a system. The sgRNA probe matches the target, and Cas12a cleaves the quenched fluorescent ssDNA reporter.	[40]
Nucleotide MALDI-TOF-MS	Genome	MTBC<br>NTM	The test can be used directly in clinical specimens and after mycobacteria culturing.	[41]
MGIT-seq	Genome	MTBC<br>NTM	The test is based on the NTM identification at the subspecies level using core genome multilocus sequence typing (cgMLST), and on prediction of macrolide and amikacin resistance directly from MGIT culture-positive broths using the MinION sequencer.	[42]

Table S1. *Cont.*

Test	Target	Bacteria	Notes	Reference
Myco-Panel (Medical & Biological Laboratories Co., Ltd., Tokyo, Japan)	*16S rRNA*, *sod*, RD1, the 16S-23S internal transcribed spacer (ITS) region, and indel A and indel B regions	*M. tuberculosis* var. *tuberculosis*, *M. tuberculosis* var. *BCG*<br>*M. avium, M. intracellulare, M. kansasii, M. abscessus* subsp. *abscessus, M. abscessus* subsp. *bolletii, M. abscessus* subsp. *massiliense, M. chelonae, M. gordonae, M. xenopi, M. fortuitum, M. szulgai, M. marinum/M. ulcerans, M. scrofulaceum, M. simiae, M. asiaticum, M. lentiflavum, M. nonchromogenicum, M. shimoidei, M. terrae, M. shinjukuense, M. mucogenicum, M. peregrinum, M. triviale, M. malmoense, M. chimaera, M. heckeshornense.*	PCR–reverse sequence-specific oligonucleotide probe using biotin-labeled specific primers, and species-specific-probe tagged bead mix. The hybridization was detected by a streptavidin-phycoerythrin conjugate. The test can be used directly in clinical specimens, and 83.1% concordant results between culture and sequencing approaches were observed.	[43]
Metagenomic	Genome	*M. tuberculosis*<br>NTM	The test was performed using bronchoalveolar lavage fluid and sputum.	[44]
3D-μPAD-LF-LAMP	LAMP target<br>IS*6110-106* – MTBC<br>IS*1245-49* – *M. avium*<br>*tuf*-92 – *M. abscessus*<br>KU-8 – *M. fortuitum*<br>ITS-4 – *M. intracellulare*	MTBC<br>*M. avium, M. abscessus, M. fortuitum, M. intracellulare, M. kansasii*	3D paper-based analytical 3D- μPAD device is incorporated with a closed lateral flow (LF) strip into a loop-mediated isothermal amplification (LAMP) device. The test can be used directly in clinical specimens and exhibits a LoD of 10 fg/reaction	[45]

	MKAN_RS12360-43 – *M. kansasii*			

ABC: ATP-binding cassette transporter; MTB: *Mycobacterium tuberculosis*; MTBC: *Mycobacterium tuberculosis* complex; LC-MS/MS: liquid chromatography–tandem mass spectrometry; MALDI-TOF-MS: matrix-assisted laser desorption time-of-flight mass spectrometry; HRM: high resolution melting curve.
